# Evolutionary Dynamics of Homophily and Heterophily

**DOI:** 10.1038/srep22766

**Published:** 2016-03-08

**Authors:** Pouria Ramazi, Ming Cao, Franz J. Weissing

**Affiliations:** 1 Faculty of Mathematics and Natural Sciences, ENTEG, University of Groningen, Nijenborgh 4, 9747 AG Groningen, The Netherlands; 2 Groningen Institute for Evolutionary Life Sciences, University of Groningen, Nijenborgh 7, 9747 AG Groningen, The Netherlands

## Abstract

Most social interactions do not take place at random. In many situations, individuals choose their interaction partners on the basis of phenotypic cues. When this happens, individuals are often homophilic, that is, they tend to interact with individuals that are similar to them. Here we investigate the joint evolution of phenotypic cues and cue-dependent interaction strategies. By a combination of individual-based simulations and analytical arguments, we show that homophily evolves less easily than earlier studies suggest. The evolutionary interplay of cues and cue-based behaviour is intricate and has many interesting facets. For example, an interaction strategy like heterophily may stably persist in the population even if it is selected against in association with any particular cue. Homophily persisted for extensive periods of time just in those simulations where homophilic interactions provide a lower (rather than a higher) payoff than heterophilic interactions. Our results indicate that even the simplest cue-based social interactions can have rich dynamics and a surprising diversity of evolutionary outcomes.

The evolution of social behaviour does typically take place in a setting where the interaction of agents is not completely at random. Ever since Hamilton^1^,^2^, much research has focused on settings where a non-random interaction structure is caused by external factors, such as in many kin-, group-, or spatially structured populations. A rich body of theory reveals that such externally induced patterns in the interaction structure has major implications for the course and outcome of social evolution^3^,^4^,^5^,^6^,^7^,^8^. More recently, theory is being developed for situations where patterns in the interaction structure are caused internally, by the behaviour of the interacting agents. Agents may, for example, terminate interactions with defectors and seek to establish interactions with cooperators. Again, many studies show that even slight deviations from a random interaction structure can have important implications, such as the emergence of cooperation in a one-shot Prisoner’s Dilemma, which would be strongly selected against in the absence of partner choice and/or partner fidelity^9^,^10^,^11^,^12^,^13^,^14^,^15^.

It is perhaps not too surprising that social evolution takes a different course if agents can choose their interaction partners on the basis of their behavioural tendencies. Here we consider a different scenario of partner choice that mainly applies to situations where agents have no cues concerning the social behaviour of the other agents in their neighbourhood. In situations like this, agents might still make their choice of interaction partner and their behaviour in an interaction dependent on some observable characteristic like a visual or olfactory cue that can be used as a marker to distinguish between (classes of) individuals. Tag-based choices of interaction partners and/or tag-based social behaviour may lead to an association of tags with particular types of behaviour and, hence, strongly affect the outcome of social interactions. For example , Hamilton^2^, argued that altruism can be an evolutionarily stable strategy if the tendency towards altruism is closely associated with a phenotypic tag and altruistic acts are only directed towards individuals that also have this tag. However, such a ‘green beard effect’ only works if the association between tag and altruism cannot easily be broken^16^.

In recent years, various models for the joint evolution of tags and tag-based behavioural strategies have been investigated^17^,^18^,^19^,^20^,^21^,^22^,^23^,^24^,^25^. Although these models tend to make similar assumptions, the conclusions based on these models are often strikingly different^24^,^26^. For two reasons, this is not too surprising. First, tag-based strategies are conditional strategies, and it is well-known in Evolutionary Game Theory that conditional strategies have a much richer evolutionary dynamics than unconditional strategies^27^,^28^,^29^,^30^. For example, the evolutionary outcome often depends on seemingly irrelevant details, such as asymmetries that have no associations with payoffs, information, or resource-holding potential^31^,^32^,^33^. As a consequence, small differences in model assumptions can result in large differences in model outcomes. Moreover, games with conditional strategies often have multiple (actually a large number of) alternative equilibria^34^,^35^, and non-equilibrium behaviour (like oscillations) is not uncommon^30^. Second, tags and tag-based behaviour are components of a signalling system. The evolutionary dynamics of signalling systems can be quite intricate^36^, in particular if the interests of senders and receivers are not fully congruent. This is exemplified by non-equilibrium behaviour in sexual selection models^37^,^38^ or the stable coexistence of multiple signal- and signal-response strategies in models for animal communication^39^.

For these reasons, we will here consider the joint evolution of tags and tag-based behaviour in a particularly simple model. This model was developed by Feng Fu and colleagues (2012)^16^, in order to investigate the evolution of homophily (the tendency to interact with others of similar type) or heterophily (the tendency to interact with others of different type). They consider a haploid asexual population where each individual has an inherited tag (like a beard colour) and an inherited tendency *p* to be homophilic. At each point in time, an individual is either homophilic (with probability *p*) or heterophilic (with probability 1 − *p*). If two individuals meet, they only interact when they are either both homophilic and, in addition, share the same beard colour, or if they are both heterophilic and, in addition, differ in beard colour. In the first case, they receive a payoff *a* > 0; in the second case, they receive a payoff *b* > 0; and in all other cases, they receive a payoff of zero. By means of sophisticated analysis, Fu and colleagues arrive at the conclusion that homophily will evolve under a wide range of conditions, even if the payoff to homophilic interactions, *a*, is considerably smaller than the payoff to heterophilic interactions, *b*. This finding might explain why homophily seems to be more common than heterophily. Yet, it is also somewhat counter-intuitive. If there are many beard colours in the population, an individual will more often encounter different-coloured than same-coloured individuals. Accordingly, there are more opportunities for heterophilic interactions, and each lost opportunity results in the lowest payoff zero.

Fu and colleagues consider the joint evolution of a large number of tags and a continuum of mixed interaction strategies (characterized by different values of the homophilic tendency *p*) through the interplay of selection, mutation, and genetic drift. Selection is rather weak in their model, resulting in an equilibrium distribution of homophilic tendencies that does not differ much from a uniform distribution. For this reason, we first investigate a simplified version of the model, which considers only the two extreme interaction strategies (pure homophily, *p* = 1, and pure heterophily, *p* = 0). It will turn out that the evolutionary outcome is remarkably different from the mixed-strategy model of Fu and colleagues. In a second step, we add a third strategy, namely indiscriminate interaction with anybody, irrespective of beard colour. We will show that this indiscriminate strategy tends to dominate both heterophily and homophily. However, depending on the parameters *a* and *b*, the evolutionary dynamics of tags and strategies can be quite intricate, and homophily or heterophily can coexist with indiscriminate interaction for extended periods of time. In a third step, we consider not only three pure strategies but a whole spectrum of mixed strategies that are characterized by two parameters (the tendency *p* to interact in case of meeting an individual with the same tag, and the tendency *q* to interact in case of different tags). Again we will find a general tendency towards indiscriminate interaction, but the joint evolution of tags and behaviour exhibits some unexpected features, such as the long-term prevalence of homophily just in those cases where the payoff to homophilic interaction is relatively low.

## Results

### Model 1: Homophily versus heterophily

We first consider a population where all individuals are either homophilic or heterophilic. Each individual has a certain tag *i*, out of *M* available ones. Individuals meet other individuals at random. If both individuals are homophilic and also have the same tag, they interact and both receive the payoff *a* > 0. If both are heterophilic and have different tags, they interact and both receive *b* > 0. In all other situations, they do not interact and receive a zero payoff. As shown in the Methods section, the expected payoff of a homophilic and a heterophilic individual can then be written as:


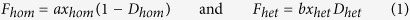


where *x*_*hom*_ and *x*_*het*_ = 1 − *x*_*hom*_ are the relative frequencies of homophilic and heterophilic individuals in the population, while *D*_*hom*_ and *D*_*het*_ denote the Gini-Simpson indices^40^ for tag diversity among homophilics and heterophilics, respectively. *D*_*hom*_ and *D*_*het*_ are given by


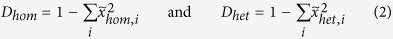


where 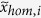 and 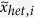 denote the relative frequency of tag *i* among homophilics and heterophilics, respectively. Tag diversity *D* varies between 0 (when only a single tag is present) and 1 − 1/*M* (when all *M* tags are present in equal frequencies).

From (1) we can conclude that the payoffs of homophilics and heterophilics are both positively frequency dependent: for a given distribution of tags, the payoff of an interaction strategy increases with the frequency of this strategy, and the payoff gets very small if an interaction strategy is rare. As a consequence, there are two types of stable equilibrium, corresponding to the fixation of the homophilic strategy or the heterophilic strategy. If the heterophilic strategy reaches fixation, the heterophilic behaviour of the population members induces negative frequency dependent selection on the tags, leading to a uniform distribution of tags. In contrast, fixation of the homophilic strategy induces positive frequency dependent selection on the tags, leading to the fixation of one of the tags. In the resulting population, the term ‘homophily’ loses its meaning, since in the absence of tag variation everybody interacts with everybody else.

**Figure 1:**
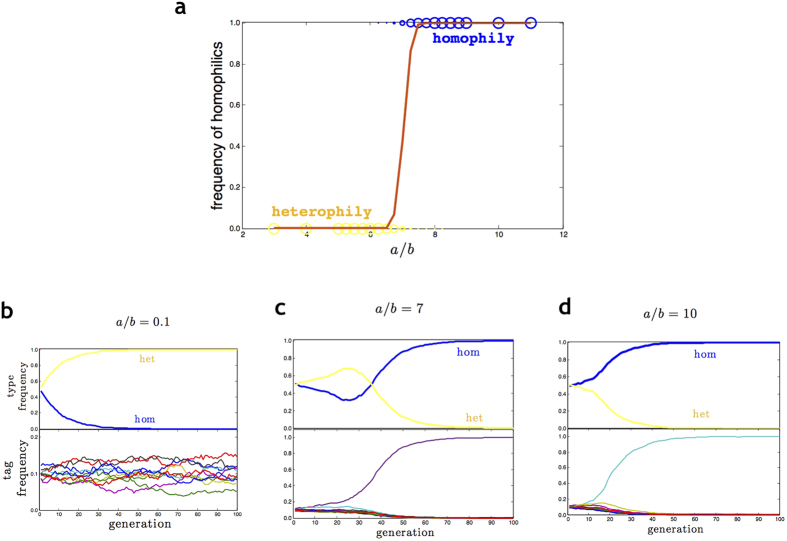
Effect of payoffs on the evolution of homophily and heterophily. (**a**) Summary of simulation outcomes for 20 combinations of the payoff ratio *a*/*b* (*a*: payoff to homophilic interaction; *b*: payoff to heterophilic interaction). Each of the 100 replicate simulations per parameter combination resulted either in the fixation of homophily (blue) or in the fixation of heterophily (yellow). The size of the circles indicates the number of times that the corresponding fixation state was achieved. The brown line indicates the mean frequency of homophilics at the end of the simulation (averaged over all 100 replicates). Homophily only evolved when *a* was considerably larger than *b*. (**b**–**d**) Representative simulation runs showing the changes in the relative frequency of homophily and heterophily (upper panels) and the associated relative tag frequencies (lower panels). (**b**) If *a*/*b* < 6.5, heterophily spreads to fixation and tag frequencies fluctuate around a uniform distribution. (**c**) If *a*/*b* = 7, either homophily or heterophily spreads to fixation, often after a period of coexistence. (**d**) If *a*/*b* > 7.5, homophily spreads to fixation; only one tag remains in the population. Parameters: *N* = 1000, *M* = 10, *b* = 1, (**b**) *a* = 0.1, (**c**) *a* = 7, (**d**) *a* = 10.

To get an impression of which of the equilibria will be more easily attained, we ran individual-based evolutionary simulations for 20 combinations of the payoff parameters *a* and *b*. Since selection depends on relative payoffs, the course and outcome of evolution are determined by the ratio *a*/*b* (rather than by the individual values *a* and *b*). Per parameter combination, 100 replicate simulations were initiated symmetrically, with an equal frequency of homophilics and heterophilics, and a uniform tag distribution. Under these conditions (*x*_*het*_ = *x*_*hom*_ and *D*_*het*_ = *D*_*hom*_ = 1 − 1/*M*), eqn (1) implies that the payoff of the homophilic strategy is smaller than or equal to the payoff of the heterophilic strategy, unless *a*/*b* > *M* − 1. This is reflected in Fig. 1, which shows that virtually all simulations with *a*/*b* ≤ 6.5 resulted in a heterophilic population with high tag diversity (see Fig. 1b), while the simulations with *a*/*b* ≥ 7.5 resulted in a homophilic population with only a single tag remaining (Fig. 1d). Only for a relatively small range of parameter values 6.5 < *a*/*b* < 7.5 both types of equilibria were attained; in these simulations it often took a while until it became clear whether homophily or heterophily prevailed in the end (Fig. 1c). Notice that the threshold value *a*/*b* ≈ 7.0 for the evolution of homophily from symmetric starting conditions is somewhat smaller than the criterion *a*/*b* > *M* − 1 = 9 for *F*_*hom*_ > *F*_*het*_. The discrepancy is explained by the fact that for *F*_*hom*_ ≈ *F*_*het*_ any tag with a slightly larger frequency than 1/*M* is subject to positive frequency-dependent selection (since it is relatively often involved in homophilic interactions), leading to an increased dominance of one tag and, as a consequence, a higher fitness of the homophilic interaction strategy.

In the main text, we only show simulations for the special case. As shown in the Supplementary Information, the same results were, *ceteris paribus*, obtained for other values of M as well.

These results of our Model 1 are in striking contrast to the findings of Fu and colleagues (2012). Also in their model, homophily is the predominant strategy whenever *a*/*b* is larger than a critical threshold *K*, but this threshold (which reflects the model parameters like population size, mutation rates, and number of tags) is substantially smaller than *M*-1 and typically smaller than 1. From the fact that homophily can be predominant even for *a* < *b* (which can happen if *K* < *a*/*b* < 1), Fu and colleagues conclude that homophily is intrinsically favoured. In our two-strategy model, we do not find any indication for such an intrinsic advantage of homophily. From Fig. 1, one might even conclude the opposite: in our model, the homophilic payoff *a* needs to be considerably larger than the heterophilic payoff *b* in order to induce the evolution of homophily.

**Figure 2:**
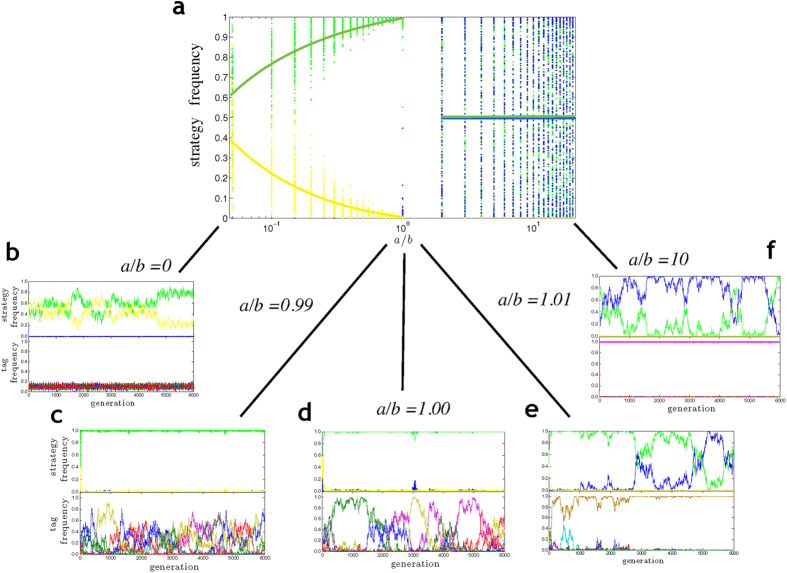
Evolution of homophily and heterophily in the presence of indiscriminate interactors. (**a**) Summary of simulation outcomes for the selection among three interaction strategies: homophily (blue dots), heterophily (yellow dots), and indiscriminate cooperation (green dots). For a range of payoff ratios *a*/*b* (*a*: payoff to homophilic interaction; *b*: payoff to heterophilic interaction) 100 replicate simulations were conducted. Dots indicate the relative frequency of the strategies after 5000 generations in a replicate; solid lines give an impression of the average outcome per parameter combination. (**b**–**f**) Representative simulation runs showing the evolutionary dynamics of interaction strategies (upper panels) and tags (lower panels). (**b**) If *a*/*b* ≪ 1, tags approach a uniform distribution; heterophily coexists with indiscriminate interaction and reaches a comparable frequency. (**c**) If *a*/*b* < 1, heterophily and indiscriminate interaction coexist, but tag frequencies exhibit large fluctuations. When *a*/*b* approaches one, heterophily only reaches marginal frequencies. (**d**) If *a*/*b* = 1, tag frequencies fluctuate largely by genetic drift. Homophily may get off the ground when one tag happens to dominate, but most of the time indiscriminate cooperation prevails. (**e**) If *a*/*b* > 1, indiscriminate cooperation rapidly becomes the dominant strategy. Tags are largely fluctuating due to genetic drift. If one tag becomes dominant, homophily gets off the ground, driving the tag to fixation. From this point onwards, homophily and indiscriminate cooperation coexist; their frequencies fluctuate due to genetic drift. (**f**) If *a*/*b* ≫ 1, the same dynamics as in (**e**) emerges, but the fixation of one of the tags occurs much more rapidly. Parameters: *N* = 1000, *M* = 10, *b* = 1, (**b**) *a* = 0, (**c**) *a* = 0.99, (**d**) *a* = 1, (**e**) *a* = 1.01, (**f**) *a* = 10.

**Figure 3:**
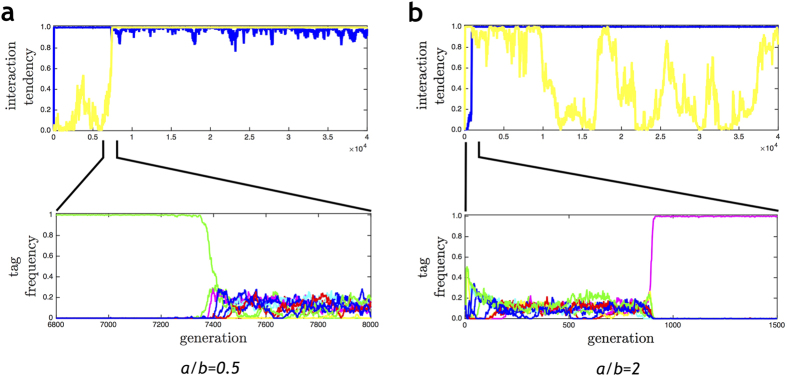
Evolution of homophilic and heterophilic tendencies. Change in the average homophilic tendency *p* (blue) and the average heterophilic tendency *q* (yellow) and the corresponding tag frequency dynamics (lower subplots) for two payoff scenarios: (**a**) *a*/*b* = 0.5, (**b**) *a*/*b* = 2. Initially all individuals have genotype (*p*, *q*) = (0, 0) (*i.e.*, they do not interact at all), but mutations with a larger value of *p* and/or *q* are rapidly selected. The two simulations exemplify two different runaway processes. In (**a**), the homophilic tendency readily reaches 1, while one tag dominates the rest. As long as only tag is present, *q* changes due to mutation and genetic drift. Once *q* reaches a threshold value (in generation 6550), rare tags are being selected, resulting in high tag diversity. In parallel, q rapidly converges to 1, leading to a population of indiscriminate interactors (*i.e.*, (*p*, *q*) = (1, 1)). In (**b**) the heterophilic tendency readily reaches 1 and the tag diversity stays at a high level. As soon as a mutation with a large value of *p* gets associated with a tag that happens to have a high frequency, homophily is selected, resulting in the convergence of *p* to 1 and the fixation of one tag. In the absence of tag diversity, the value of *q* does not matter anymore; accordingly it follows a random walk due to mutation and genetic drift.

Fu and colleagues classify an evolved population as ‘homophilic’ if the average value of the homophilic tendency, 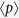, is larger than 0.5. The figures in their paper (e.g. Figs 1, 2, 3 in^41^) reveal that for most parameter combinations the deviation of 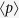 from 0.5 is minute (say, 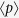 = 0.51 or 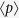 = 0.52). Our version of the model has the advantage that the evolutionary outcome is much more clear-cut: for any parameter combination, either homophily or heterophily will spread to fixation. Which of the two strategies prevails depends as much on the initial conditions as on the payoff parameters.

### Model 2: Adding indiscriminate interaction to the model

In the basic version of the model of Fu and colleagues, at each decision moment an individual has to be either homophilic (with probability *p*) or heterophilic (with probability 1−*p*). Since the payoff is zero when no interaction takes place (and positive in case of an interaction), it does not seem reasonable to reduce one’s number of interactions by only interacting with individuals with specific tags. For this reason, we extend our model by a third strategy, indiscriminate interaction. Indiscriminate individuals are open to interaction with any tag. If such an individual meets an individual with the same tag, an interaction yielding payoff *a* results, unless the other individual is heterophilic; if it meets an individual with a different tag, an interaction yielding payoff *b* results, unless the other individual is homophilic. If a meeting does not result in an interaction, the payoff is zero.

Again, we conducted 100 replicate simulations for a broad spectrum of payoff ratios *a*/*b*. The results are summarized in Fig. 2a. Although the simulation outcomes per parameter combination vary a lot, a clear pattern emerges. If the homophilic payoff *a* is smaller than the heterophilic payoff *b*, the homophilic strategy disappears, and the two other strategies converge to a polymorphism, where the relative frequency of the indiscriminate strategy (green) is larger than the relative frequency of the heterophilic strategy (yellow). If *a* is larger than *b*, the heterophilic strategy disappears and the indiscriminate strategy coexists with the homophilic strategy (blue) in – on average – equal frequencies. We will now consider the two types of outcome in more detail.

The relation between *a* and *b* determines whether the interaction strategies induce positive or negative frequency dependent selection on the tags. When *a* > *b*, more frequent tags have a selective advantage because individuals with such tags will be more frequently involved in the more profitable homophilic interactions than individuals with less frequent tags. As a consequence, one of the tags will eventually spread to fixation (see Fig. 2e,f). Once the diversity of tags has disappeared, indiscriminate and homophilic individuals behave in exactly the same way. Accordingly, none of the two strategies has a selective advantage, and they coexist in a neutral manner, that is, their relative frequency is determined by genetic drift. When *a* < *b*, less frequent tags have a selective advantage because individuals with such tags will be more frequently involved in more profitable heterophilic interactions. Now, selection on tags is negatively frequency dependent; all tags remain in the population and the tag frequencies are quite similar (see Fig. 2b). Since the payoff associated with homophilic interactions is low, the homophilic interaction strategy rapidly disappears from the population. One might expect that heterophilics have a selective disadvantage as well, since they reject same-tag interaction partners (yielding a payoff of zero) while indiscriminate interactors receive payoff *a* > 0 when interacting with same-tag individuals. As shown in the Methods, indiscriminate interactors do indeed have a higher expected payoff than heterophilics if the tag distribution is the same for both types of interaction strategy. Yet, as shown in Fig. 2 the heterophilic strategy can stably coexist with the indiscriminate strategy. This can be explained as follows. For any given tag *i*, indiscriminate individuals with this tag have a selective advantage over the heterophilic individuals with this tag (since they also get payoffs from same-tag interactions), but this advantage is small if the frequency of tag *i* is small (since meetings of same-tag individuals are rare in this case). As a consequence, the heterophilic strategy becomes statistically associated with the rarer tags. Rarer tags, however, provide a higher fitness, since they are less often involved in the less profitable same-tag meetings. Due to its association with rare tags, the heterophilic strategy can achieve the same fitness as the indiscriminate strategy: indiscriminate interactors get both types of payoff *a* and *b*, but relatively often get *a*; heterophilics can only get payoff *b*, but they get this higher payoff more frequently than indiscriminate interactors. Due to this mechanism, heterophilics can stably coexist with indiscriminate interactors, and this coexistence is to a large part mediated by selection.

### Model 3: A continuum of mixed strategies

After having considered two models with only two or three interaction strategies, we now have a closer look at the mixed-strategy model considered by Fu and colleagues (2012) in their Supplementary Information. Basically, the model is as before, but now individuals are no longer either homophilic or heterophilic or indiscriminate all the time. Instead, each individual is endowed with two heritable tendencies: a tendency *p* to interact with individuals with the same tag (homophilic tendency) and a tendency *q* to interact with individuals with a different tag (heterophilic tendency). The tendencies *p* and *q* both evolve in the course of time (see Methods for details).

As exemplified by the simulations in Fig. 3, there are essentially two different outcomes. When *a* < *b* (Fig. 3a), the tags remain highly polymorphic and both the homophilic tendency *p* (blue) and the heterophilic tendency *q* (yellow) converge to the maximal value 1. Accordingly, the population evolves to a state of indiscriminate interaction. When *a* > *b* (Fig. 3b), *p* and *q* also converge to 1, followed by the fixation of one of the tags. Once tag diversity has been lost, the heterophilic tendency *q* has no effect anymore; accordingly, the changes in *q* are no longer governed by selection, but by mutation and random genetic drift.

Although the outcome of all simulations of the mixed-strategy model is highly predictable, the transient dynamics to this outcome has some interesting features. First, notice that initial phase of the evolutionary dynamics, which is characterized by the rapid convergence of either *p* or *q* to the maximal value 1, is somewhat counterintuitive: if the homophilic payoff *a* is larger than the heterophilic payoff *b*, the heterophilic tendency *q* converges more rapidly to 1 (Fig. 3b), while the homophilic tendency *p* converges more rapidly to 1 if *a* is smaller than *b* (Fig. 3a). Second, we observed a long (but transient) period of homophily (*p* = 1, *q* = 0) only in those cases where the homophilic payoff *a* was smaller than *b* (Fig. 3a). This occurred when a mutation with a larger value of *p* (and a still small value of *q*) got associated with a tag of relatively high frequency. Such an association can trigger a period of runaway selection, where the tag spreads since it profits from the homophilic tendency of its carriers, while an increase in *p* is strongly selected due to the prevalence of the tag. As shown in the Methods, the fact that *a* < *b* implies that only strategy combinations (*p*, *q*) with a large discrepancy between *p* and *q* (i.e., a large value of *p*/*q*) can become associated with a frequent tag. As a result, *p* rapidly converges to 1 and the associated tag spreads to fixation. This is, however, not the end of the story. In a single-tag population, the heterophilic tendency *q* is subject to genetic drift. As shown in the Methods, rare tags will be selected as soon as *q* exceeds the threshold value 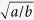. In the simulation in Fig. 3a (where 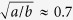), this happens in generation 7380. From this time onwards, tag diversity is rapidly recovered, and the heterophilic tendency *q* converges to the maximal value 1. The homophilic tendency *p* also stays close to 1, but selection on *q* is much stronger than selection on *p*.

A similar runaway process takes place in case of *a* > *b* (Fig. 3b). In the initial phase, a spectrum of *p*-values can coexist in the population, keeping tag variation at a relatively high level. In a situation like this, a large value of *q* (and only a rather large one) can spread, thereby inducing negative frequency dependent selection on the tags. Repeated mutations at the *q*-locus induce the rapid convergence of *q* to 1, corresponding to a population of heterophilics. However, after some time (about 900 generations in the simulation in Fig. 3b) a high value of *p* gets associated with a tag that happens to have a larger-than-average frequency. If *p* is sufficiently large, this induces positive frequency dependent selection on the tag, leading to the fixation of the tag. Now a rapid succession of invasion invents leads to the convergence of *p* to 1.

Despite the transient dynamics described above, the long-term outcome of evolution is always indiscriminate interaction; either because *p* and *q* have both converged to 1 (if *a* < *b*, Fig. 3a) or because one tag eventually spreads to fixation due to positive frequency dependent selection (if *a* > *b*, Fig. 3b).

## Discussion

Homophily, the tendency to preferentially interact (and cooperate) with individuals of similar phenotype, is widely observed in nature^42^,^43^,^44^ and, in particular, in human societies^45^,^46^,^47^. The model of Fu and colleagues (2012) may be viewed as a minimal model for the evolutionary analysis of homophily and heterophily. The original analysis of this model^41^ revealed that, quite remarkably, homophily is the predominant interaction strategy under a wide variety of conditions, including situations where the payoff associated with homophilic interactions is smaller than the payoff associated with heterophilic interactions. Accordingly, the model seems to provide a simple and general explanation for the ubiquity of homophily in nature.

However, three aspects of the study of Fu and colleagues are not fully convincing. First, this study does not provide a sound intuitive understanding of the obtained results. Intuitively, one would have expected that in this simple model heterophily predominates more easily than homophily, since in the presence of many tags there are many more opportunities for heterophilic interactions than for homophilic interactions. Second, the effects observed by Fu *et al*.^41^ are very weak. The equilibrium distribution of homophilic tendencies differs only very little from a uniform distribution, and homophily is only marginally more frequent than heterophily. Third, and perhaps most importantly, the study of Fu *et al*.^41^ largely neglects the possibility that individuals interact indiscriminately, thereby increasing the number of potential interactions. Since each encounter between individuals that does not result in an interaction is associated with the lowest possible payoff (zero), engaging in indiscriminate interaction seems a dominant strategy. In fact, an additional analysis in the Supplementary Information of Fu *et al*.^41^ reveals that indiscriminate interaction dominates homophily and heterophily (their Fig. S13 for *a* > 0 and *b* > 0), but from the main text of their article it becomes clear that the authors do not view this as a counter-argument against the predominance of homophily in their model.

In order to find out whether homophily does indeed arise under very mild conditions, we re-investigated the minimal model of Fu *et al*.^41^ under conditions where the evolutionary outcome is more clearly determined by natural selection (and less by stochastic factors like mutation and genetic drift). We found that even in this simple model the co-evolution of tags and tag-based behaviour has intricate dynamics with interesting and sometimes counter-intuitive facets. For example, heterophily stably coexists with indiscriminate interaction (Fig. 2, *a*/*b* < 1) despite the fact that for *any* given tag *i* the *i*-bearing heterophilics have a lower fitness than the *i*-bearing indiscriminate interactors. In the simulations of Model 3, the population was often homophilic for an extended initial period if heterophilics had a higher payoff (Fig. 3a), while it was heterophilic for an initial period if homophilics had a higher payoff (Fig. 3b). All these results can be explained by reciprocal feedbacks between tags and tag-based interaction strategies: the fitness of interaction strategies strongly reflects the distribution of tags; while the distribution of tags reflects natural selection on the tags, which can be positively or negatively frequency dependent, depending on the distribution of interaction strategies.

Concerning the question whether the prevalence of homophily can be explained on the basis of a minimal model, we arrive at the opposite conclusion than Fu and colleagues. Homophily did only emerge as an evolutionary outcome when homophily and heterophily were the only interaction strategies (Model 1); in all other cases, homophily was ousted from the population (unless only a single tag remained in the population, a situation where the difference between homophily and indiscriminate interaction becomes irrelevant). Even in Model 1, the domain of attraction of homophily was very small (unless the payoff advantage *a*/*b* of homophily was very large). Moreover, the dominance of homophily was associated with the fixation of a single tag (Fig. 1d), which is again a situation where ‘tag-based behaviour’ loses its meaning. In fact, homophily would rapidly disappear from a single-tag population, if we would make the reasonable assumption that tag discrimination involves some costs.

The fixation of a single tag could not occur if tags are not inherited, but randomly assigned at birth. In a scenario like this, indiscriminate interaction outcompetes both homophily and heterophily in Models 2 and 3. In Model 2, homophily and heterophily are selected against for all values of *a*/*b* and coexistence with indiscriminate interaction can no longer occur. In Model 3, *p* and *q* both rapidly converge to 1, without a transient initial period of homophily or heterophily. In Model 1, homophily and heterophily are still alternative attractors. However, homophily will only evolve if its initial frequency is very high. Assuming a uniform distribution of tags (implying *D*_*hom*_ = *D*_*het*_ = 1 − 1/*M*), eqn (1) reveals that homophily has a selective advantage if, and only if


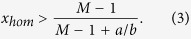


For example, homophily will only spread in the special case *a* = *b* if *x*_*hom*_ > 1 − 1/*M*. Hence, also in a model without tag inheritance, homophily only evolves under restrictive conditions.

From all this, we conclude that other factors must also play an in important role in explaining the prevalence of homophily. One such factor may be spatial structure: in a spatially structured population tag-based interaction strategies may either strengthen or weaken the spatial correlation of phenotypes and strategies in a population, with major implications for the course and outcome of evolution. In fact, spatial structure has been incorporated in several models for the evolution of tag-based interaction strategies, with a high diversity of evolutionary outcomes^19^,^48^,^49^,^50^,^51^,^52^,^53^,^54^,^55^. A second factor is the nature of the interaction, which on purpose is kept very simple in the model of Fu and colleagues. Homophily may have a crucial role in coordinating the agents’ behaviour in a coordination game^56^,^57^,^58^ or, more generally, in achieving a favourable outcome in ambiguous situations^59^,^60^,^61^. For example, most social interactions have a large number of possible (Nash) equilibrium solutions^27^,^28^,^33^. In case of repeated games, the ‘Folk Theorem’ of game theory^35^ states that *any* ‘reasonable’ outcome can be realized by a Nash equilibrium. In a situation like this, the problem is not to find one of the potential solutions, but to coordinate behaviour in such a way that the same solution is selected by all interaction partners^34^,^62^. Perhaps, homophily has evolved to resolve such coordination problems, which universally occur in all kinds of social interactions.

## Methods

### Individual-based simulations

We consider a haploid asexual population with a fixed size of *N* = 1000 individuals. Each individual has one of *M* heritable tags (where *M* = 10 in all simulations) and a heritable interaction strategy. The model is event-based. Whenever an event occurs, one randomly chosen individual is removed from the population and replaced by the descendant of a population member. The probability that a given individual *k* is the parent of this descendant is proportional to *k*’s expected payoff when meeting a random population member (which for Models 1 and 2 is given by equations (5), (6) and (7) below). The descendant inherits both the tag and the interaction strategy from its parent. However, with probability 0.001 the parent’s tag mutates into any of the *M* tags (with equal probability), and with probability 0.001 the parent’s interaction strategy is affected by mutation. In Models 1 and 2, the interaction strategy (homophily, heterophily, indiscriminate) mutates into a randomly chosen alternative strategy. In Model 3, the interaction strategy (*p*, *q*) mutates by adding an amount *ε* to either *p* or *q*, where the mutational step size *ε* is normally distributed with mean 0 and standard deviation 0.05. Populations were initiated with a uniform distribution of tags and an equal frequency of all interaction strategies in Models 1 and 2; in Model 3, all individuals were initialized with (*p*, *q*) = (0, 0). In our model, a ‘generation’ corresponds to *N* = 1000 events, i.e., the average lifespan of an individual.

### Mathematical analysis

The relative frequencies of homophilics, heterophilics, and indiscriminate interactors carrying tag *i* are denoted by *x*_*hom*,*i*_, *x*_*het*,*i*_ and *x*_0,*i*_, respectively. The relative frequencies of all homophilics, all heterophilics, and all indiscriminate interactors are denoted by 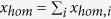, 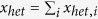, and 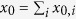, respectively. The relative frequency of tag *i* in homophilics, heterophilics, and indiscriminate interactors are therefore given by


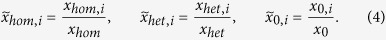


The expected payoff (=fitness) of a homophilic, a heterophilic, and an indiscriminate individual are obtained from


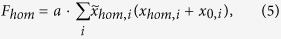



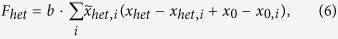



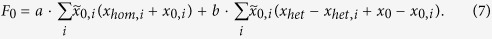


In the absence of indiscriminate interactors (Model 1), the fitness of homophilics and heterophilics can be written in the form of Eq. (1):


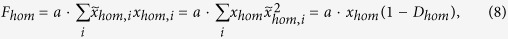



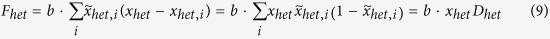


where 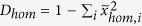 and 
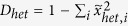
 denote the Gini-Simpson^35^ for tag diversity among homophilics and heterophilics, respectively.

In the general case (Model 2), the fitness difference between an indiscriminate and a heterophilic individual is given by


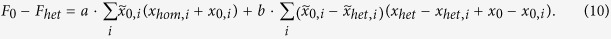


This implies that the fitness difference is positive if indiscriminate interactors and heterophilics have the same tag distribution. In other words, indiscriminate interaction provides a fitness advantage in this case. From this we conclude that the stable coexistence of indiscriminate interactors and heterophilics in Fig. 2 (indicating that *F*_0_ = *F*_*het*_) is only possible because the tag distribution differs between the two interaction strategies.

To explain the results of Model 3, we give an intuitive explanation in the main text that can be given a formal underpinning as follows. Assume that the population is monomorphic for the interaction strategy (*p*, *q*) and that only the two tags 1 and 2 are present, with relative frequency *x*_1_ and *x*_2_. Then the fitness difference between the two tags is given by


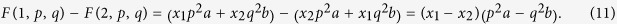


Hence the more frequent tag has a fitness advantage if 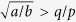, while the less frequent tag has a fitness advantage if 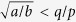.

## Additional Information

**How to cite this article**: Ramazi, P. *et al*. Evolutionary Dynamics of Homophily and Heterophily. *Sci. Rep.* **6**, 22766; doi: 10.1038/srep22766 (2016).

## Supplementary Material

Supplementary Information
