# Supplementary material for: Evolutionary Dynamics of Homophily and Heterophily
Source: Sci Rep. 2016 Mar 8;6:22766. doi: 10.1038/srep22766 (PMC4782132; doi:10.1038/srep22766)
Supplement: Supplementary Information [file srep22766-s1.pdf]

Evolutionary Dynamics of Homophily and Heterophily (Supplementary Information)

Pouria Ramazi, Ming Cao, Franz J. Weissing

Three figures have been presented in the main text, each corresponding to one of the models. The explanation of the patterns described in the main text do not depend on the number of tags M. We therefore do not expect changing M would affect our results and conclusions. Here we confirm this expectation by reproducing the three figures in the main text (which were based on $M = 10$) for the values $M = 5$ and $M = 20$.

Model 1

$M = 5$:

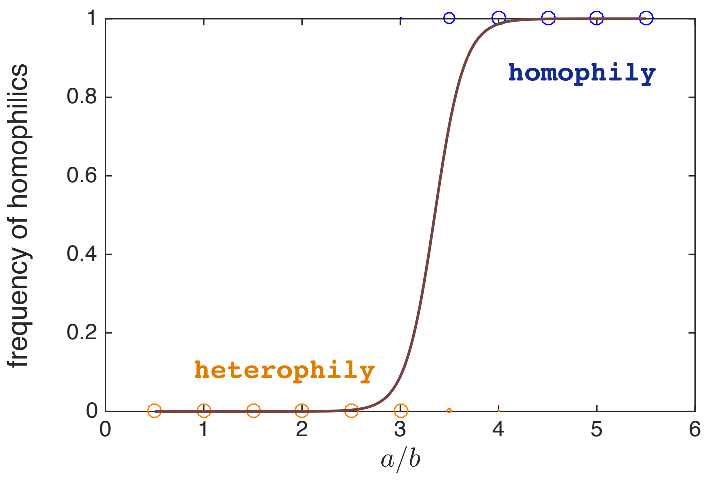

$M = 10$:

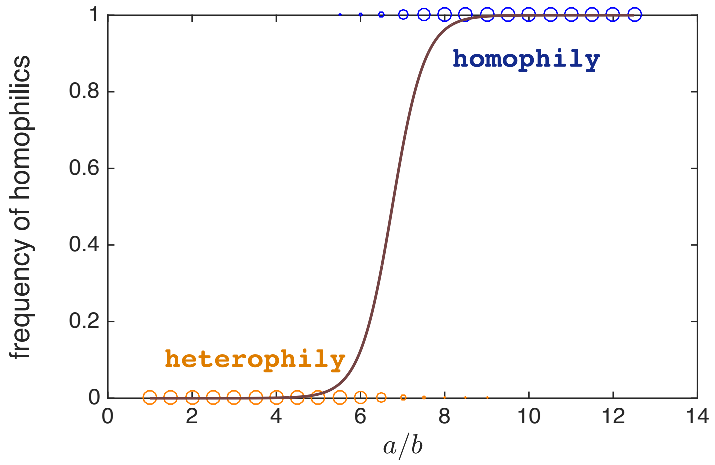

$M = 20$:

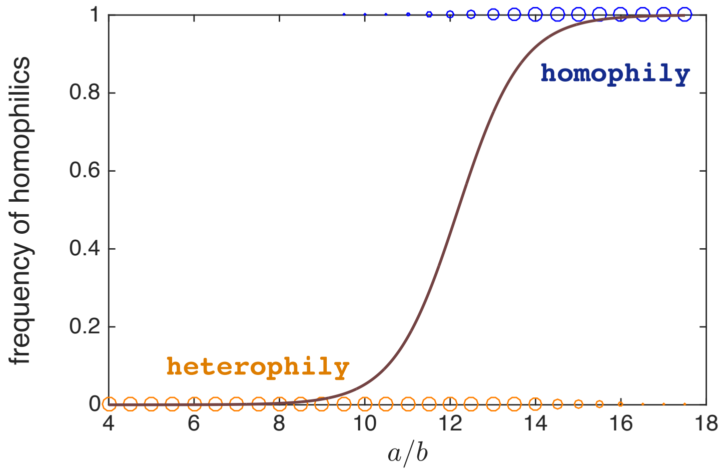

Figure S1. Effect of the number of tags(M) on the evolutionary outcome in Model 1. The figure depicts the number of fixation with respect to homophily and heterophily for various values of the quotient a/b of the payoff parameters. Figure conventions are as in Fig. 1 in the main text. Qualitatively, the pattern of fixation is not affected by parameter M: For $a/b > M - 1$, homophily went to fixation in all cases and for $a/b < M/2$, heterophily went to fixation in all cases. For intermediate values, fixation of either of the two takes place.

Model 2

$M = 5$:

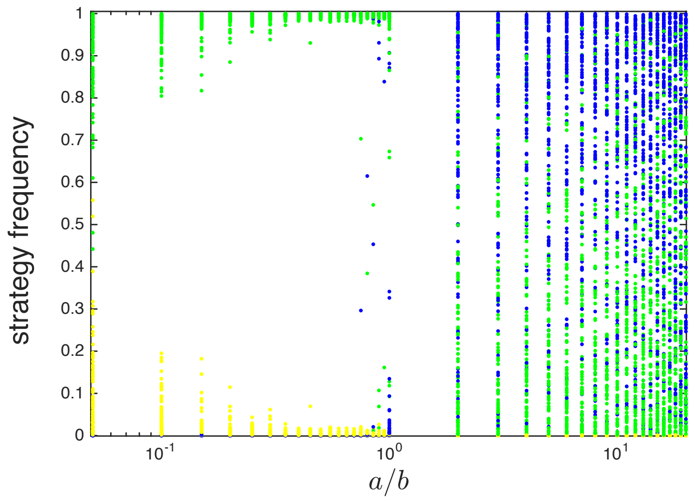

$M = 10$:

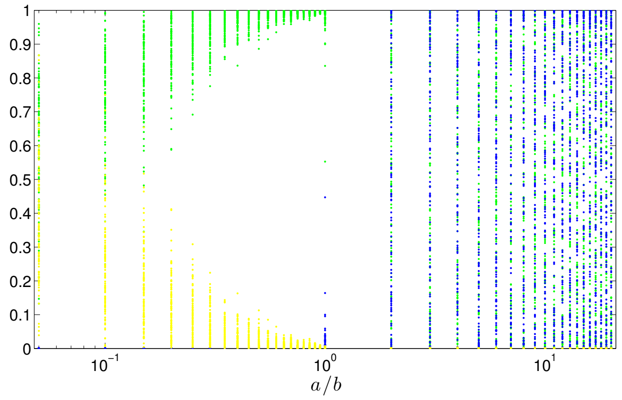

$M = 20$:

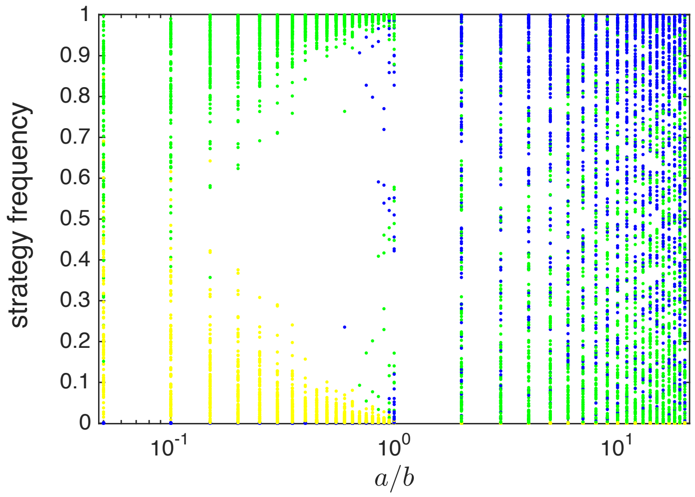

Figure S2. Effect of the number of tags(M) on the evolutionary outcome in Model 2. The figure depicts the evolution of homophily and heterophily in the presence of indiscriminate interactors. Figure conventions are as in Fig. 2 of the main text. The qualitative pattern of the simulations is not affected by M: As before, for $a/b < 1$, homophily disappears, and the two other strategies converge to a polymorphism, where the relative frequency of the indiscriminate strategy (green) is greater than the relative frequency of the heterophilic strategy (yellow). For $a/b > 1$, heterophily disappears, and a single tag goes to fixation. As a consequence, indiscriminate cooperation induces identical behavior as homophily (blue) implying that the frequency of both strategies changes due to genetic drift.

Model 3

$M = 5$:

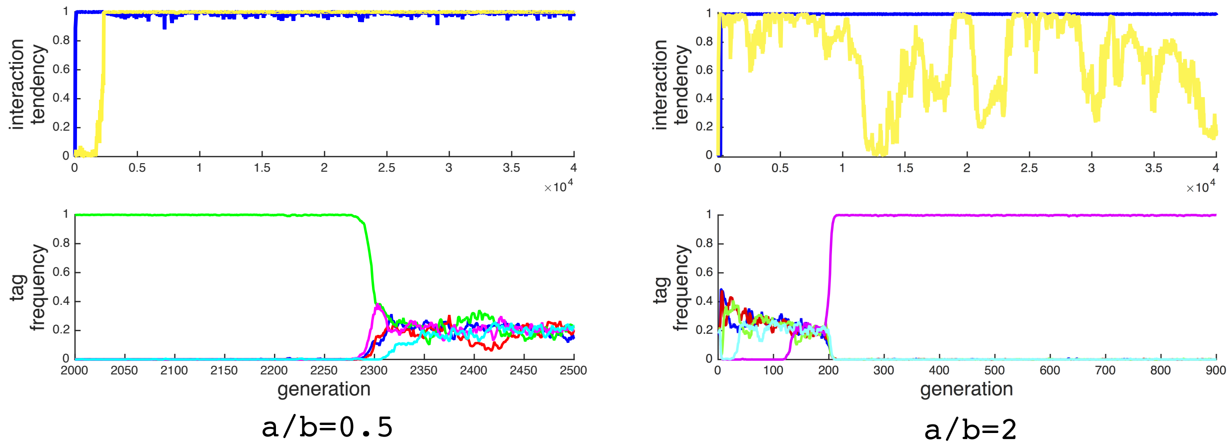

$M = 10$:

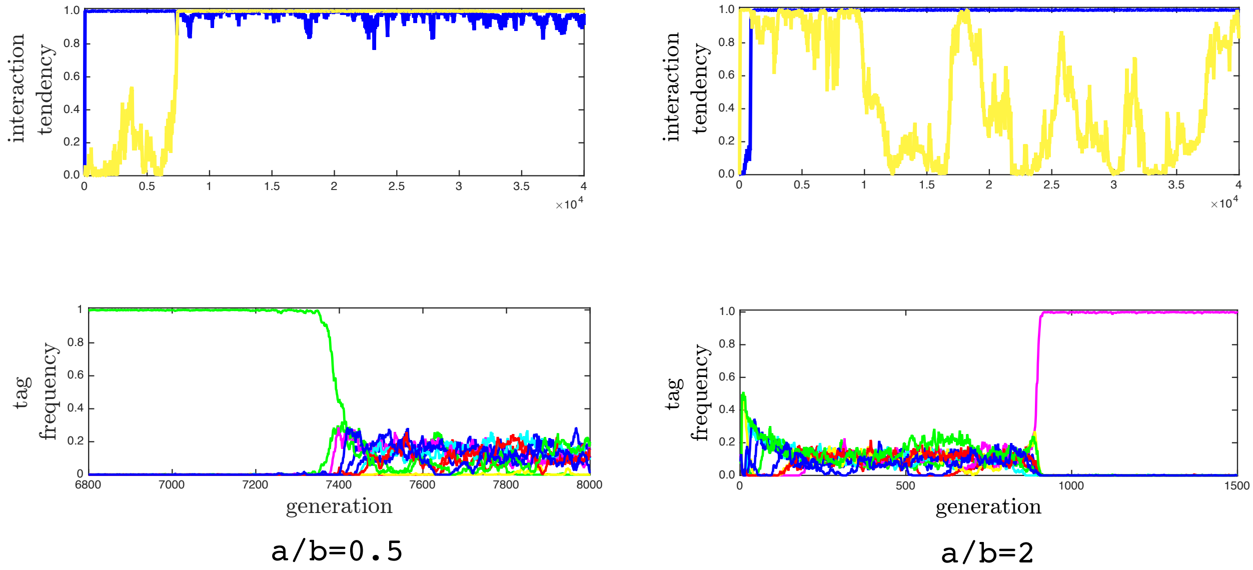

$M = 20$:

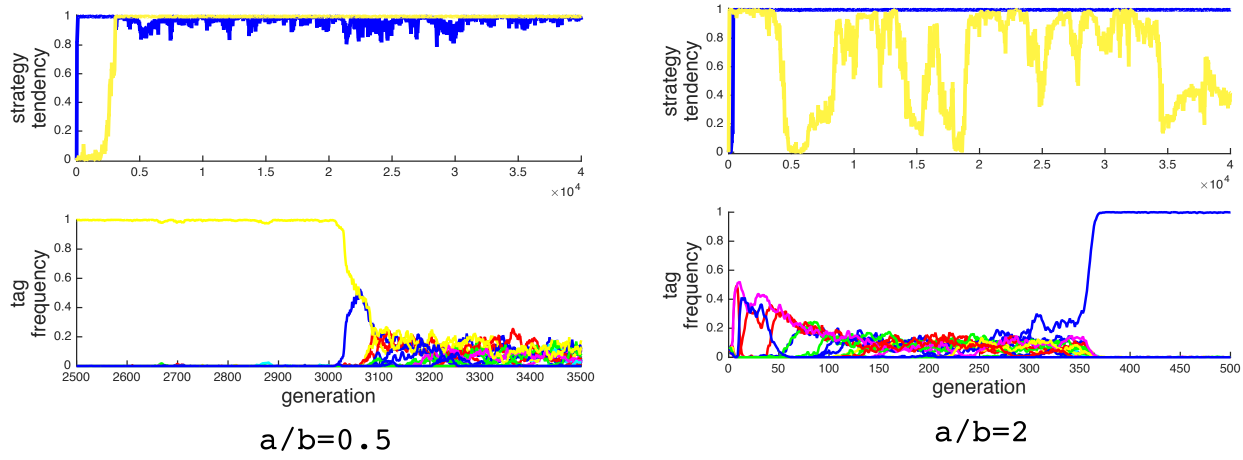

Figure S3. Effects of the number of tags(M) on the evolutionary outcome in Model 3. The figure depicts the evolution of the homophilic tendency p (blue; the tendency to interact with a same-tag individual) and the heterophilic tendency q (yellow; the tendency to interact with a different-tag individual). The qualitative pattern of the simulations is not affected by M: As before, for $a < b$, the tags remain highly polymorphic and both p and q converge to the maximal value 1. Accordingly, the population converges to a state of indiscriminate interaction. When $a > b$, p and q also converge to 1, followed by the fixation of one tag. Once tag diversity is lost, the heterophilic tendency q has no effect anymore; accordingly, the changes in q are no longer governed by selection, but by mutation and random genetic drift.
